# Radiotherapy-activated NBTXR3 nanoparticles modulate cancer cell immunogenicity and TCR repertoire

**DOI:** 10.1186/s12935-022-02615-w

**Published:** 2022-06-03

**Authors:** Audrey Darmon, Ping Zhang, Julie Marill, Naeemunnisa Mohamed Anesary, Jordan Da silva, Sébastien Paris

**Affiliations:** Nanobiotix, Paris, France

**Keywords:** CD8+ T cells, Immunopeptidome, NBTXR3, Radioenhancer, Radiotherapy, TCR

## Abstract

**Background:**

Radiotherapy is a powerful and widely used technique for the treatment of solid tumors. Beyond its ability to destroy tumor cells, it has been demonstrated that radiotherapy can stimulate the anti-tumor immune response. Unfortunately, this effect is mainly restricted to the irradiated lesion, as tumor control outside the treated field (called the ‘abscopal effect’) is rarely obtained. In addition, many pro-tumoral factors prevent this anti-tumor immune response from being sustained and efficient. We previously reported that radiotherapy-activated NBTXR3 produced a significant CD8-dependent abscopal effect in immunocompetent mice bearing CT26.WT tumors, while radiotherapy failed to generate such a response.

**Methods:**

To identify the mechanisms that may explain this response, we evaluated the capacity of radiotherapy-activated NBTXR3 to modulate the immunogenicity of tumor cells by analysis of immunogenic cell death biomarkers and immunopeptidome sequencing. In vivo, we analyzed treated tumors for CD4+, CD8 + and CD68 + cell infiltrates by immunohistochemistry and digital pathology and sequenced the T cell receptor (TCR) repertoire in both treated and untreated distant tumors.

**Results:**

We showed that NBTXR3 activated by radiotherapy both increased immunogenic cell death biomarkers and modulated the immunopeptidome profile of CT26.WT cells. Immunohistochemistry analysis of treated tumors revealed a significant increase in CD4+, CD8 + and CD68 + cell infiltrates for NBTXR3 activated by radiotherapy group, compared to radiotherapy. We also measured significant modifications in TCR repertoire diversity in the radiotherapy-activated NBTXR3 group, both in treated and distant untreated tumors, compared to radiotherapy alone.

**Conclusions:**

These results indicate that radiotherapy-activated NBTXR3 can act as an effective immunomodulator, modifying tumor cell immunogenicity and impacting the lymphocyte population.

**Graphical Abstract:**

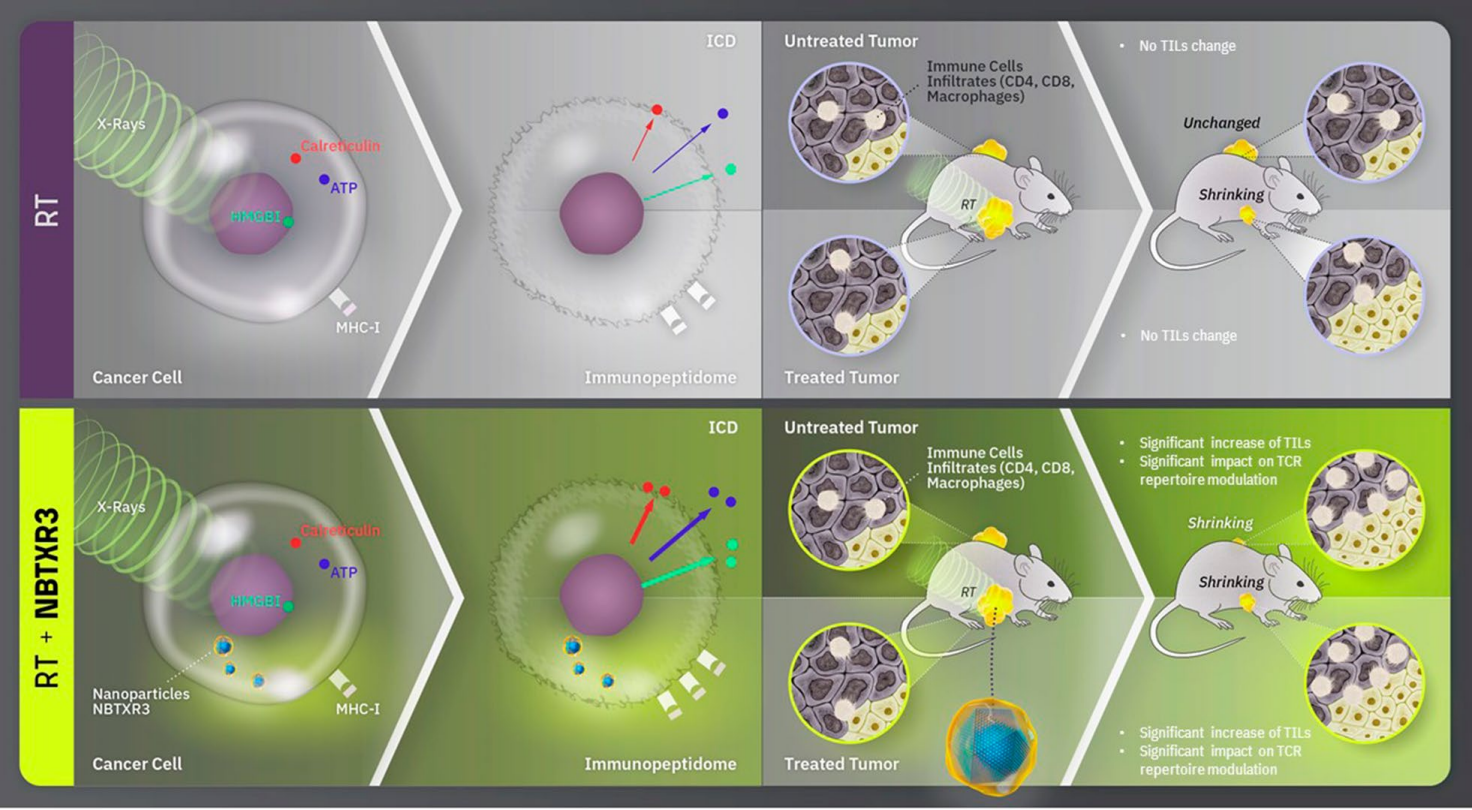

**Supplementary information:**

The online version contains supplementary material available at 10.1186/s12935-022-02615-w.

## Introduction

For over a century, radiation therapy (RT) has been used to treat solid tumors. It is one of the major pillars of the therapeutic arsenal in the fight against cancer. Currently, about half of patients receive RT during their cancer care journey [1]. The primary mode of action of RT is based on physical interactions between X-ray photons and the atoms crossed, generating direct damage to molecules such as DNA, producing double-strand DNA breaks (DSB). RT also generates indirect damage to cellular components through the production of reactive oxygen species (ROS), which can result in additional DSB. If this damage is too overwhelming for the cell to repair, it will trigger the cell’s death.

However, the effects of RT go beyond simply cell destruction. Numerous studies have reported that RT can modulate several biological pathways closely associated with the anti-tumor immune response (ATIR) [2–7]. RT can modulate cancer cell immunogenicity through MHC class I expression [5, 8], improving the presentation of tumor-associated antigens (TAA) and tumor-specific antigens (TSA) at the cancer cell membrane [9], which allows the adaptive immune system to better recognize and kill malignant cells. Unfortunately, the anti-tumoral immunomodulatory effects of RT are often too limited or counterbalanced by various pro-tumor elements, such as myeloid derived suppressor cells (MDSCs) and regulatory T cells (Treg) [10], to prevent an efficient and/or sustained ATIR from eradicating all cancer cells. In addition, the effects of RT are limited to the irradiated tumor area, and the production of an abscopal effect (when RT not only shrinks the targeted tumor but also leads to the shrinkage of untreated distant tumors) is rarely obtained. Only 46 reported cases have been identified from 1969 to 2014 [11]. Nonetheless, the immunomodulatory effects of RT make it attractive to combine with checkpoint inhibitors like anti-PD-1 or anti-PD-L1. Preclinical data support this rationale and paved the way for clinical developments [12]. However, despite encouraging clinical results, the response rate remains low, with inconsistent results among tumor types, [13, 14], thus the effectiveness of this approach needs to be improved.

In this context, the combination of RT with new technological approaches seems essential to produce an efficient ATIR. NBTXR3 (functionalized hafnium oxide nanoparticles), a first-in-class radioenhancer designed to interact with ionizing radiation to increase RT energy deposition inside tumor cells, has demonstrated its anti-tumor capacity in preclinical settings in a large panel of human cancer models [15–17]. The clinical efficacy of NBTXR3 was demonstrated in patients with locally advanced Soft Tissue Sarcoma in the randomized controlled Phase II/III Act.in.Sarc study (NCT02379845) [18]. We also demonstrated the capacity of radiotherapy-activated NBTXR3 (NBTXR3 + RT) to significantly improve cGAS-STING pathway activation in the human colon cancer cell line HCT116 [19], a cellular response important for the activation of antitumor immunity [20]. In mice bearing mouse colon cancer CT26.WT tumors, NBTXR3 + RT triggered a potent anti-tumor CD8 + T cell immune response as well as a significant abscopal effect, drastically improving tumor control and survival [21]. Recently, it has been demonstrated that NBTXR3 + RT combined with anti-PD-1 significantly enhanced local tumor control, abscopal effect, and survival of mice bearing lung tumors resistant to anti-PD-1 treatment [22]. Taken together, these results demonstrate the enhanced immunomodulatory capacity of RT-activated NBTXR3. However, information at the cancer cell level which could explain these results is lacking. In this article, we explored the impact of NBTXR3 + RT treatment on tumor cell immunogenicity and the potential modifications of TCR repertoire diversity to better understand the underlying mechanisms that could explain the effectiveness of NBTXR3 + RT in activating the ATIR.

## Materials and methods

### Cells and reagents

The mouse colon cancer CT26.WT (#CRL-2638) and human colon cancer HCT116 (#CCL-247) cell lines were purchased from the American Type Culture Collection (ATCC, USA). The human glioblastoma 42-MG-BA cell line was purchased from the (Deutsche Sammlung von Mikroorganismen und Zellkulturen (DSMZ, Germany). Cells were cultivated according to provider’s recommendations. NBTXR3 (Nanobiotix, France) is a sterile aqueous suspension of functionalized HfO_2_ nanoparticles with a size centered on 50 nm, bearing a negative surface charge in aqueous solution at pH 6–8 [15].

### Mice

Six-week-old BALB/cJrj female mice (Janvier Labs, France) were maintained under pathogen-free conditions in the animal facility at Gustave Roussy Institute (Villejuif, France). All animal experiments were carried out in compliance with French and European laws and regulations (European Directive 2010/63 EU). The local institutional animal ethics board and the French Ministry of Research approved all mouse experiments (permission numbers: 2016_031_4340 and 2016_128_8343).

### Irradiation

For in vitro calreticulin exposure analysis, radiation was delivered using an X-ray generator at 125 kV (CellRad, Faxitron, USA) or at 320 kV (X-RAD 320, Precision X-Ray, USA). For immunopeptidome analysis, cells were irradiated with the X-ray generator at 320 kV. For in vivo assays, tumor irradiation was performed with a 200 kV irradiator (NDI 226 X-ray, Varian, USA). Selective irradiation of mice tumors was performed by the interposition of a lead shield, allowing full protection of the rest of the body, including proximal lymph nodes.

### Calreticulin exposure analysis

NBTXR3 was added overnight to HCT116, 42-MG-BA and CT26.WT cells before delivering the radiation dose. NBTXR3 concentration, irradiation dose, irradiation source, and the number of individual experiments for each cell line are reported in Additional file 1: Table S2. For analysis of cell-surface exposure of calreticulin (Ecto-CALR), cells were harvested using Accutase (#A6964, Sigma-Aldrich, France) and fixed in 0.25% paraformaldehyde (#11,586,711, Thermo-Fisher, France) 24 h after irradiation. A rabbit anti-calreticulin primary antibody (#Ab4, Abcam, France) was added to cells and visualized using an anti-rabbit Alexa 488 (#Ab150077, ThermoFisher, France). Cells were counter-stained with Live/Dead fixable Far Red Dead Cell stain kit (#L34974, Thermo-Fisher, France) to exclude dead cells and Ecto-CALR was measured by cytofluorometry (Accuri C6+, Becton Dickinson, France).

### Immunopeptidome analysis

CT26.WT cells were either treated or not treated with 400 µM of NBTXR3. The following day, cells were either irradiated with 4 Gy or did not receive radiation. After one day, cells were harvested by scraping, centrifuged, and the dry cell pellets were stored at − 80 °C. Immunoaffinity chromatography was used to capture MHC class I complexes using W6/32 Ab-linked resin. MHC-associated peptides were analyzed by LC-MS/MS by Cayman Chemical (USA). Cell pellets were thawed on ice, then lysed at 20.10^6^ cells/ml of lysis buffer, incubating for 30 min on ice. The resin was washed and combined with clarified lysates by gentle rotation at 4 °C overnight. After washing, peptides were eluted then concentrated and desalted using solid-phase extraction with an Empore C18 plate. For mass spectrometry analysis sample preparation, peptides were loaded directly and eluted using 80/20 acetonitrile/water (0.1% TFA). Eluted peptides were lyophilized and reconstituted in 0.1% TFA. For mass spectrometry (MS) analysis, peptides (50% per sample) were analyzed by nano LC/MS/MS using a Waters NanoAcquity system interfaced to a ThermoFisher Fusion Lumos mass spectrometer. Peptides were loaded on a trapping column and eluted over a 75 μm analytical column at 350 nL/min; both columns were packed with Luna C18 resin (Phenomenex). A 2 h gradient was employed. The mass spectrometer was operated using a custom data-dependent method, with MS performed in the Orbitrap at 60,000 FWHM resolution and sequential MS/MS performed using high-resolution CID and EThcD in the Orbitrap at 15,000 FWHM resolution. All MS data were acquired from m/z 300–800. A 3s cycle time was employed for all steps.

### In vivo experiments

For TCR repertoire analysis, 3.10^5^ CT26.WT cells were subcutaneously injected into both flanks of mice on the same day. Once the tumor had grown (70 to 130 mm^3^), mice were randomized to the different groups. A volume of NBTXR3 suspension (or vehicle) corresponding to 25% of the baseline tumor volume was injected intratumorally into the right flank tumor only (i.e., the left flank tumor was untreated). After 24 h, right tumors were irradiated with 4 Gy per fraction for 3 consecutive days. For immunohistochemistry and digital pathology analysis, the same workflow as for TCR repertoire analysis was used, except that CT26.WT cells were injected only in the right flank. Length (L) and width (W) of tumors were measured with a digital caliper. Tumor volumes were calculated using the formula (LW^2^/2). The animals were euthanized by cervical dislocation.

### TCR repertoire analysis

All mice were sacrificed 72 h after the last fraction of RT and both treated and untreated tumors were immediately excised, and frozen at − 80 °C before TCR repertoire analysis. DNA from each tumor was extracted then analyzed using the immunoSEQ™ mmTCRβ kit for mouse T-cell receptor repertoire characterization, by sequencing of the mouse TCRβ locus at survey level sequencing resolution (Adaptive Biotechnologies). To this aim, amplification, and sequencing of CDR3 regions in the TCRβ locus using multiplex PCR amplification across the VDJ junction of rearranged TCRβ locus was performed for each sample.

### Immunohistochemistry and digital pathology analysis

All mice were sacrificed 5 days after the last fraction of RT and the tumors were immediately excised then fixed. For each tumor, 3 slices of 4 μm from FFPE blocks (first third, middle, and last third of the tumor) were put on the same slide and slices were stained using specific antibodies raised against CD4 (#50,134-R0001, Interchim, France), CD8 (#AB203035, Abcam, France), or CD68 (#AB125212, Abcam, France) on a Ventana Discovery XT autostainer and stained by haematoxylin/eosin/safran on a Leica ST5020 multistainer. For digital pathology analysis, each stained slide was scanned with Aperio AT Turbo x40 (Leica, Germany) by Excilone (France).

## Statistical analysis

Normality distribution of values was assessed by Shapiro-Wilk normality test. Immunohistochemistry (IHC) analyses were analyzed by Mann-Whitney test. For TCR repertoire, analysis with normal distribution was analyzed by one-way ANOVA while non-normal distribution was analyzed by Mann-Whitney test. A *p* value < 0.05 was considered statistically significant. The GraphPad Prism 8 v.8.2.1 software was used for graph plotting and biostatistics.

## Results

### NBTXR3 improves calreticulin exposure

**Fig. 1:**
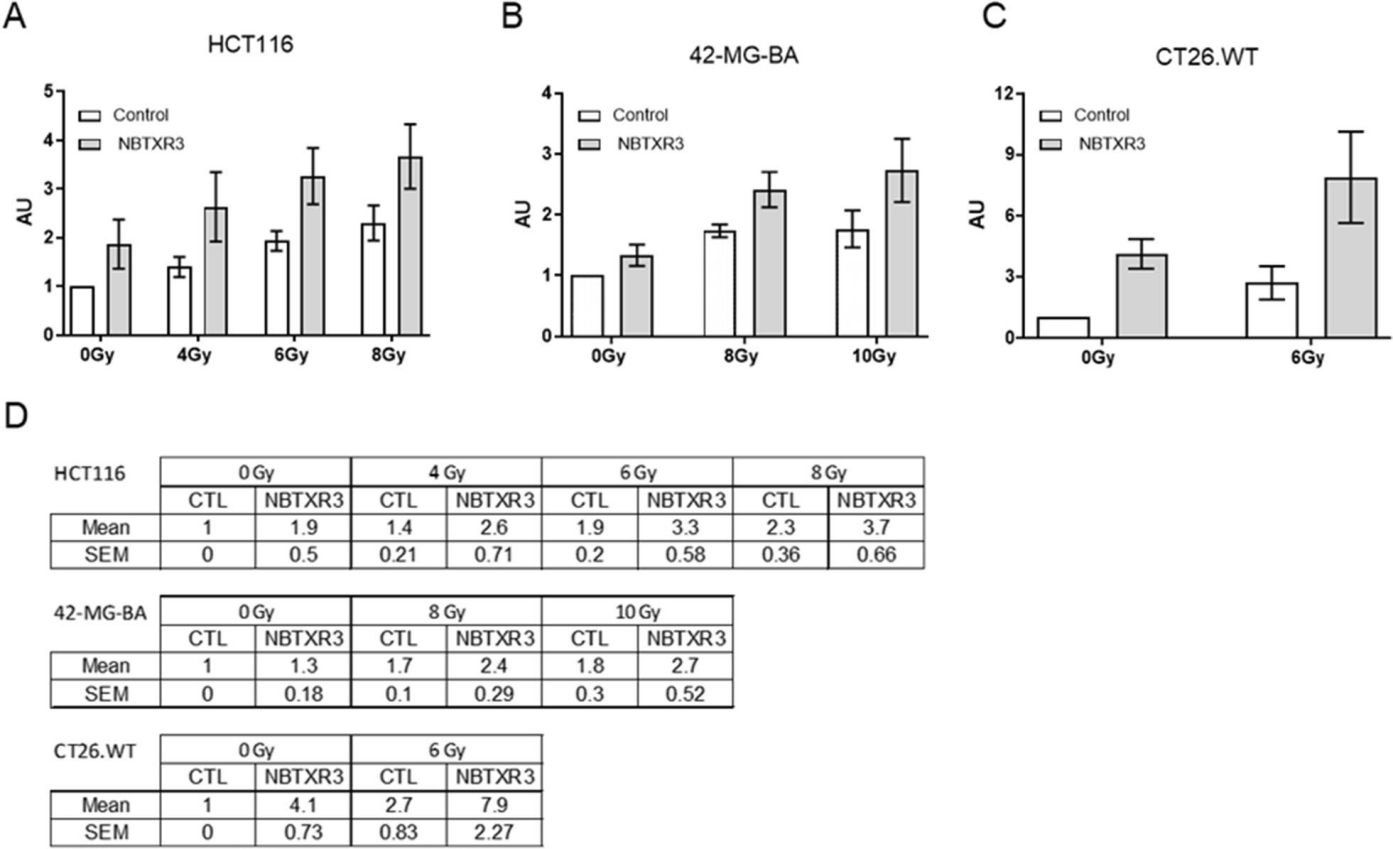
NBTXR3 Improves calreticulin exposure. Flow-cytometry analyses of the Ecto-CALR signal acquired 24 h post-treatment by RT, for (**A**) HCT116, (**B**) 42-MG-BA and (**C**) CT26.WT cells. HCT116, 42-MG-BA and CT26.WT cells were treated overnight respectively with 800µM, 100 µM or 400µM of NBTXR3, then irradiated. The next day, cells were stained for Ecto-CALR and fluorescence was determined by flow-cytometry. Presented data were obtained from at least three independent experiments (n ≥ 3). Data are represented as mean fold increase ± SEM compared to unirradiated control cells. **D** Detailed fold-increase

To assess the impact of NBTXR3 + RT on calreticulin exposure at the cell surface membrane, we irradiated HCT116, 42-MG-BA, and CT26.WT cells treated with or without NBTXR3, then analyzed Ecto-CALR by flow cytometry (Fig. 1A–C). RT induced a marked increase in Ecto-CALR in the three tested cell lines. The fold increases of calreticulin exposure for RT ranged from 1.9 ± 0.5 to 2.3 ± 0.36 for HCT116, 1.7 ± 0.1 to 1.8 ± 0.3 for 42-MG-BA, and 2.7 ± 0.83 for CT26.WT, compared to untreated control (Fig. 1D). The same result profile was obtained with NBTXR3 + RT, but with higher intensity compared to RT (Fig. 1A–C). The fold increases varied from 2.6 ± 0.71 to 3.7 ± 0.66 for HCT116, 2.4 ± 0.29 to 2.7 ± 0.52 for 42-MG-BA, and 7.9 ± 2.27 for CT26.WT (Fig. 1D). Despite an enhanced Ecto-CALR expression obtained with NBTXR3 + RT, compared to RT, there was no significant difference between the two treatments (Fig. 1A–C). A non-significant increase in Ecto-CALR was also observed in cells treated with NBTXR3 without RT.

### NBTXR3 modulates the immunopeptidome

**Fig. 2:**
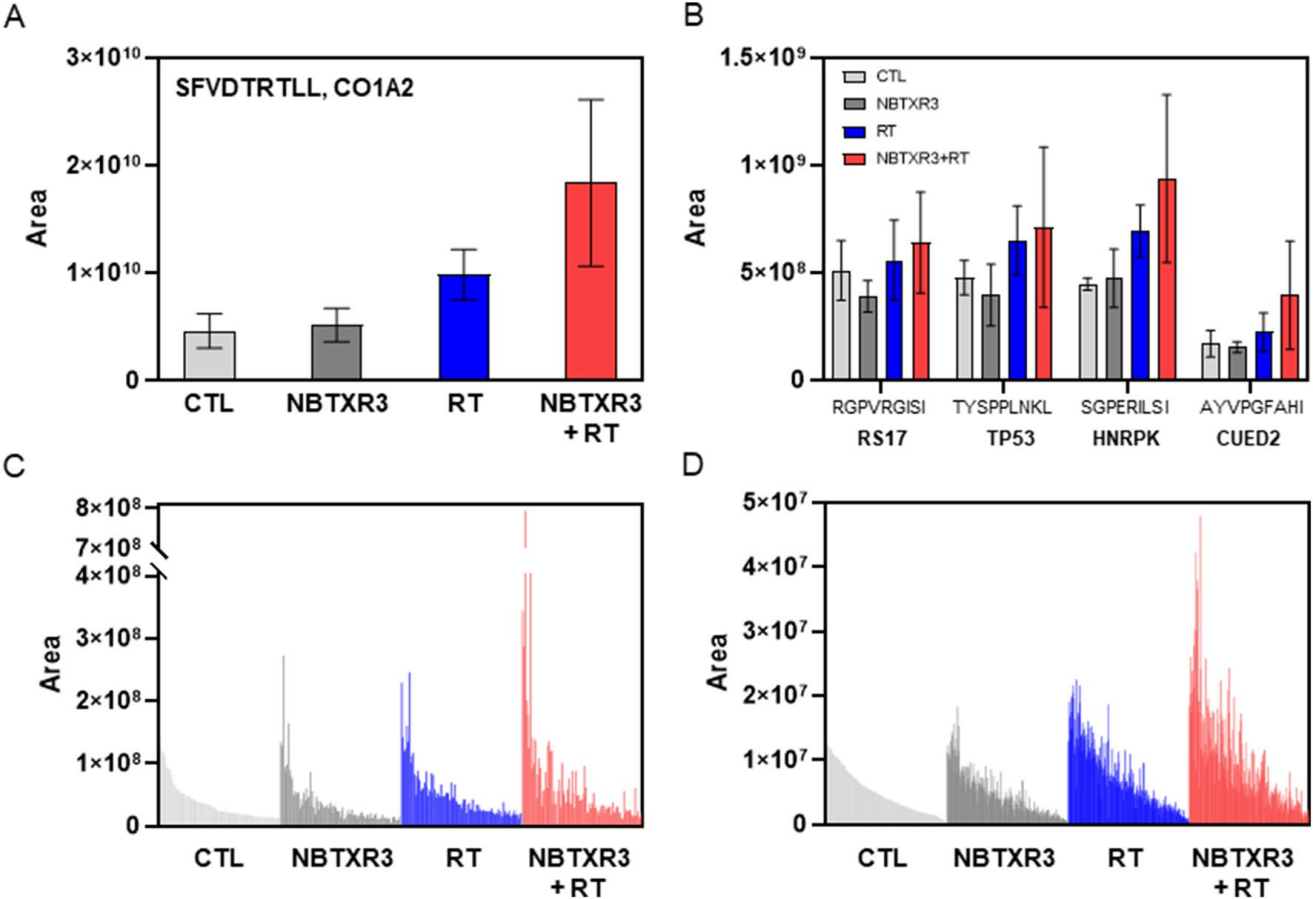
Immunopeptidome profiles obtained by LC/MS/MS analysis. CT26.WT cells were treated overnight with 400µM of NBXTR3, then irradiated with a single dose of 4 Gy. The next day, cells were collected by scraping. MHC-I complex was isolated from 20.10^6^ cells then peptides were eluted and analyzed by LC/MS/MS. Only peptides present in all independent experiments and all groups were presented (Total number = 331) and ranked according to the mean area measured for control group. **A** Variation of SFVDTRTLL peptide area, according to the tested conditions. This peptide was the most abundant, whatever the condition considered. The sequence of this peptide is related to CO1A2 (Collagen alpha-2(I) chain). **B** Variation of area for RGPVRGISI, related to RS17 (40 S ribosomal protein S17), TYSPPLNKL, related to TP53 (Cellular tumor antigen p53), SGPERILSI, related to HNRPK (Heterogeneous nuclear ribonucleoprotein K) and AYVPGFAHI peptides areas, related to CUED2 (CUE domain-containing protein 2), according to the condition tested. Area variation of peptides (**C**) #6 to #100 and (**D**) #101 to #331, according to the condition tested. Data of independent experiments (n = 3) are represented as the mean of area ± SEM

**Fig. 3:**
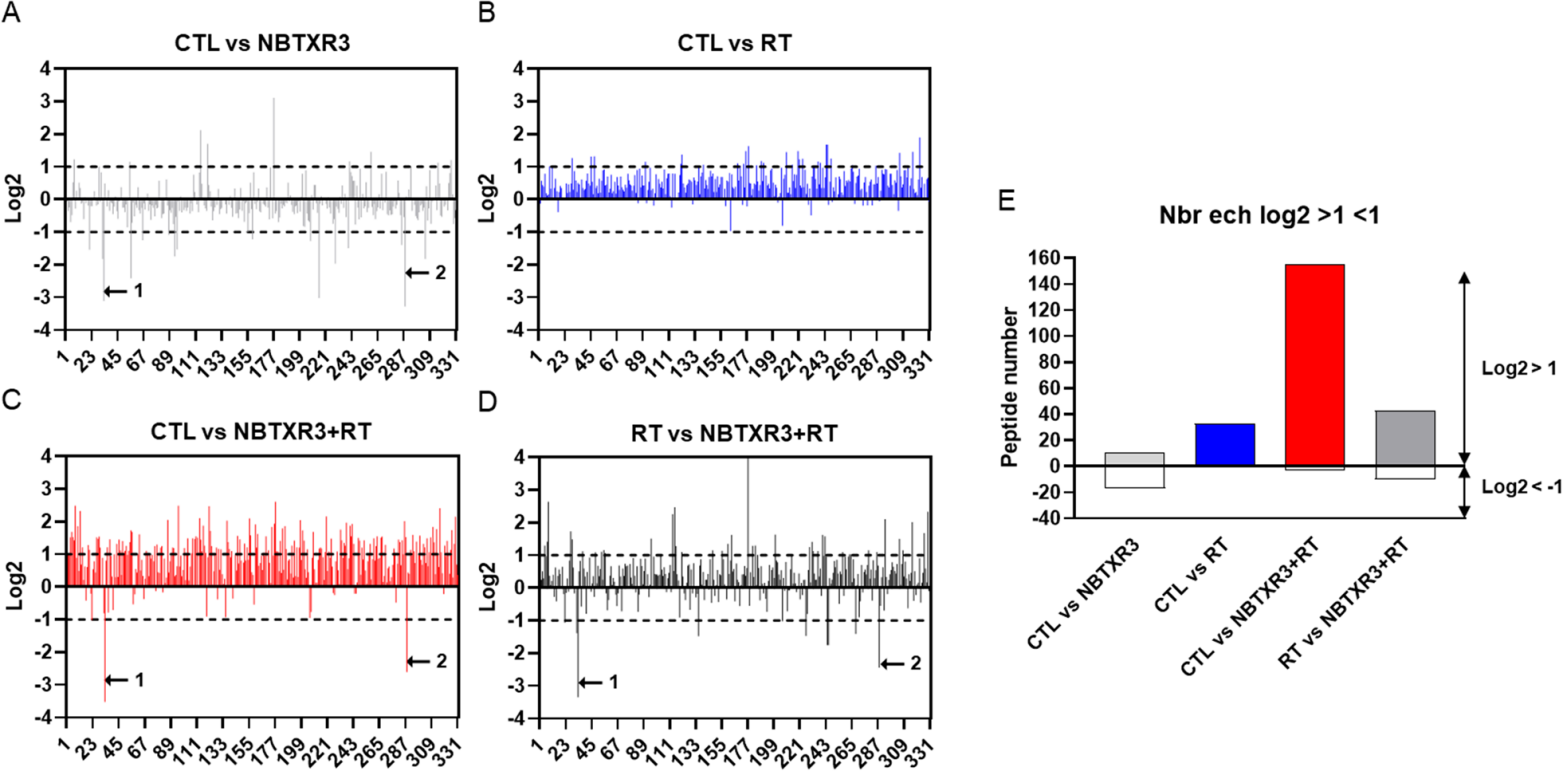
Log2 comparison of immunopeptidome profiles. For each peptide, area value variation was expressed in Log2 scale: (**A**) CTL vs. NBTXR3, (**B**) CTL vs. RT and (**C**) CTL vs. NBTXR3 + RT groups. **D** Area value variation of RT vs. NBTXR3 + RT group, expressed in Log2 scale. **A**, **C**, **D**, ←1 indicates MGPLKKDRI peptide, and ← 2 indicates SYALSRHDV peptide. **E** Number of peptides with Log2 > 1 or < − 1, according to the tested condition

The immunopeptidome plays an important role in tumor cell identification and destruction by CD8 + cytotoxic T lymphocytes. We postulated that NBTXR3 + RT could differentially modify cancer cell immunogenicity through modulation of their immunopeptidome, compared to RT alone. To validate this hypothesis, CT26.WT cells were treated under various conditions and the resulting immunopeptidomes were compared. A total of 331 epitopes (or peptides) systematically detected in each independent experiment and in each condition were considered for further analysis. LC/MS/MS analysis revealed that the vast majority of them (80.4%) had a size of 9 amino acids and > 99% had a size comprised between 8 and 12 amino acids (Additional file 1: Fig. S1). This result is consistent with the expected size of peptides eluted from MHC-I [23]. These peptides were derived from 295 different proteins (Additional file 1: Table S1). However, we reported the presence of two peptides exclusively detected in cells treated by NBTXR3 + RT: IYSEVATLI from the protein Pericentriolar material 1 (PCM1) and SGPATHDI from the protein Pleckstrin homology domain-containing family G member 2 (PKHG2). No other ‘group-specific’ peptide was found among the three other groups. The peak areas of each peptide (which reflect their abundance) were ranked in decreasing value, using the untreated control cells as reference (Fig. 2). As depicted in Fig. 2, across all treatments, the most abundant peptide was SFVDTRTLL, derived from Collagen alpha-2(I) chain (CO1A2) (Fig. 2A), followed in by RGPVRGISI from 40 S ribosomal protein S17 (RS17), TYSPPLNKL from Cellular tumor antigen p53 (TP53), SGPERILSI from Heterogeneous nuclear ribonucleoprotein K (HNRPK) and AYVPGFAHI from CUE domain-containing protein 2 (CUED2) (Fig. 2B), then peptides 6 to 331 (Fig. 2C–D). To evaluate the impact of treatments on the abundance of peptides, we converted the variation of area values into Log2 values ​​between NBTXR3, RT or NBTXR + RT, and the untreated control cells (CTL) (Fig. 3A–C). For the following analyses, we considered peptides only displaying variations of Log2 > 1 or < – 1, corresponding to a two-fold increase or decrease of the peptide abundance, respectively. Compared to CTL, the addition of NBTXR3 only slightly modified the immunopeptidome profile, compared to the control (Fig. 3A). Nonetheless, NBTXR3 increased the abundance of 11 peptides and decreased abundance of 17 peptides (Fig. 3E). In stark contrast, the abundance of almost all peptides was improved for RT (Fig. 3B), compared to CTL. The increased abundance of 33 peptides (around 10% of total peptides analyzed), and the absence of peptide with a Log2< – 1, reflects this augmentation (Fig. 3E). Remarkably, NBTXR3 + RT treatment greatly improved the abundance of peptides (Fig. 3C). Compared to untreated cells (CTL), 155 peptides doubled their abundance (46.8% of total peptides analyzed), approximately 4.7 times more than RT alone. In addition, 3 peptides have Log2< –1. Among them, the abundances of MGPLKKDRI from 60 S ribosomal protein L3 (RL3) and SYALSRHDV from Solute carrier family 35 member E1 (S35E1), were greatly reduced. Intriguingly, the abundance of these peptides was also considerably decreased by the addition of NBTXR3, but not with RT alone, suggesting an NBTXR3-related modulation. Finally, we compared the Log2 profile of RT vs. NBTXR3 + RT (Fig. 3D). As expected, because of the global enhancement of peptide abundance observed for RT, the number of peptides with Log2 > 1 for RT vs. NBTXR3 + RT was smaller than vs. CTL. Nonetheless, 43 peptides exhibit a log2 > 1 with RT vs. NBTXR3 + RT, and 10 with a log2< – 1 (Fig. 3E). As expected, MGPLKKDRI and SYALSRHDV peptide abundances were again found to be drastically decreased.

**Fig. 4:**
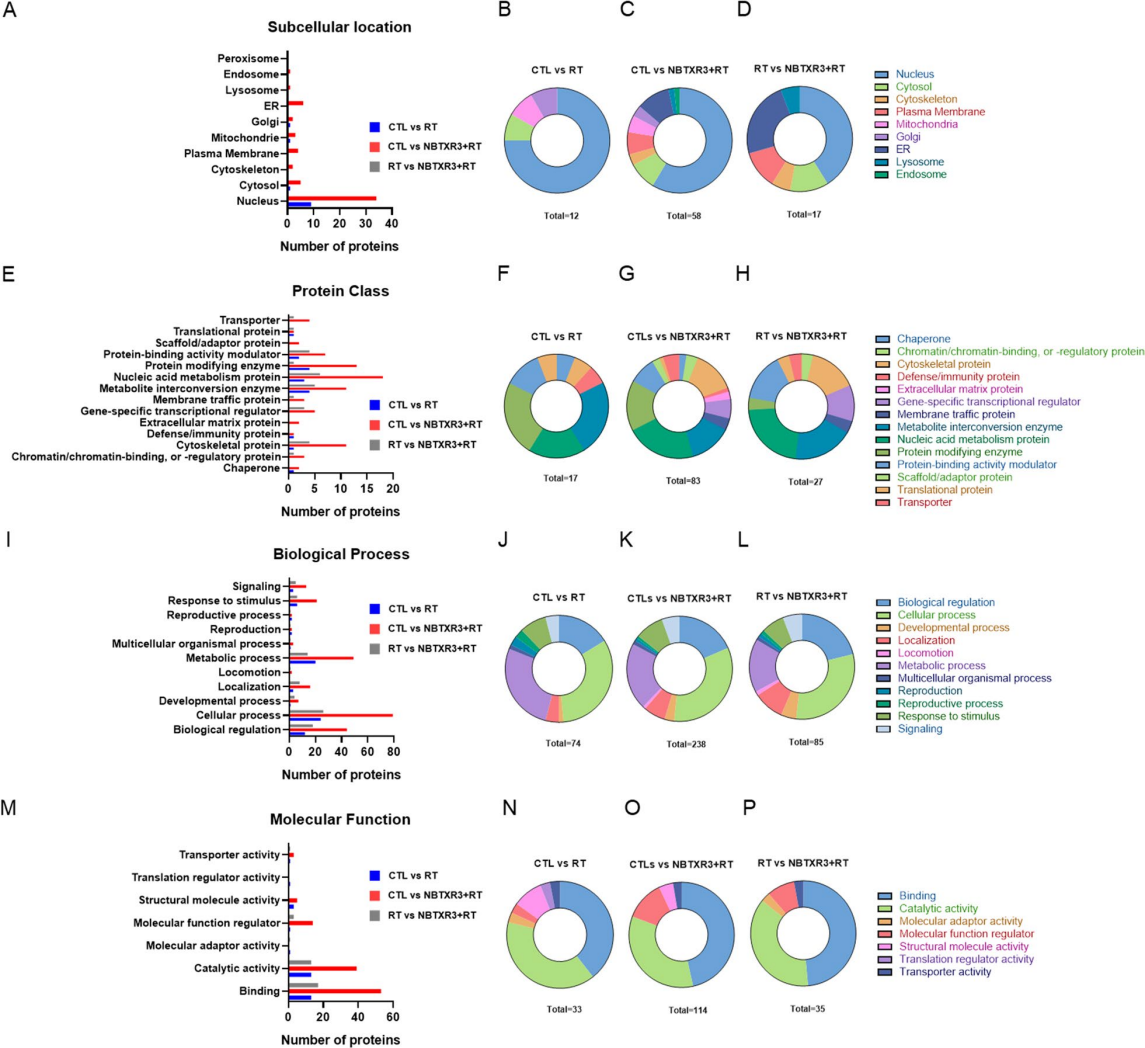
Analysis of epitopes origin. Bioinformatic analysis of (**A–D**) subcellular location, (**E–H**) protein classes, (**I–L**) biological processes and (**M–P**) molecular functions of proteins identified from peptides in the immunopeptidome (with Log2 > 1), according to the treatment considered and baseline comparator. **A, E, I, M** number of proteins found in each category. Donut representations of the percentage of each category for (**B, F, J, N**) CTL vs. RT, (**C, G, K, O**) CTL vs. NBTXR3 + RT and (**D, H, L, P**) RT vs. NBTXR3 + RT

After internalization, NBTXR3 nanoparticles are not disseminated homogenously throughout cell cytoplasm but form clusters containing a variable number of nanoparticles and were never observed in the nucleus [15–17, 22]. This heterogeneous distribution prompted us to verify if NBTXR3 + RT could favor (or limit) the proportion of some peptides, according to protein subcellular location. To evaluate this possibility, we focused our analyses on peptides of the groups RT and NBTXR3 + RT and RT vs. NBTXR3 + RT displaying a Log2 > 1. We identified the subcellular location of corresponding proteins, based on information collected on Uniprot Knowledgebase (GO-Cellular component) (Fig. 4A–D) [24]. We considered peptides were derived from proteins with a unique known intracellular location. In this context, it can be seen very clearly that most of these peptides derived from nucleus-related proteins both for RT and NBTXR3 + RT (Fig. 4A). We can also observe that this proportion seems greater for RT (75%) than for NBTXR3 + RT (58.6%) (Fig. 4B–C). This apparent difference could be explained by the presence of peptides whose original proteins were located in cellular components present in NBTXR3 + RT but not observed for RT (i.e., cytoskeleton, plasma membrane, ER, lysosome, endosome, peroxisome). Note that if these categories were no longer considered in the NBTXR3 + RT group analysis, the proportions thus obtained were greatly similar to RT alone (Additional file 1: Fig. S2). For RT vs. NBTXR3 + RT comparison, we can see that proteins of nuclear origin were overrepresented, as well as ER proteins (Fig. 4D). The results obtained were fairly comparable for RT and NBTXR3 + RT when the same analysis was performed by considering proteins coming from one or more cellular components (Additional file 1: Fig. S3).

We then wanted to determine whether the proportions of certain classes of proteins (Fig. 4E–H), biological processes (Fig. 4I–L), or molecular functions (Fig. 4M–P) could have been modulated differently depending on the treatment considered. To perform these studies, proteins that produced peptides of the immunopeptidomes with an abundance of Log2 > 1 were analyzed using the Protein ANalysis THrough Evolutionary Relationships (PANTHER) classification system [25, 26]. For protein class analysis, most of these peptides were derived from metabolite interconversion enzyme, nucleic acid metabolism protein and protein modifying enzyme-related proteins for RT and NBTXR3 + RT. For NBTXR3 + RT, the cytoskeletal proteins were also fairly represented (Fig. 4E). As for cellular component analysis, these proportions were modulated for NBTXR3 + RT by the emergence of peptides whose original protein class were not shown for RT alone (i.e., chromatin/chromatin-binding, or -regulatory protein, extracellular matrix protein, gene-specific transcriptional regulator, membrane traffic protein, scaffold/adaptor protein and transporter). Note that if these categories were no longer considered for the NBTXR3 + RT analysis, the percentage obtained for nucleic acid metabolism protein in this group became fairly more pronounced (28.12%) compared to RT alone (17.6%) (Additional file 1: Fig. S4). For RT vs. NBTXR3 + RT comparison, we can see that cytoskeletal protein, metabolite interconversion enzyme, nucleic acid metabolism protein, and protein-binding activity modulator protein classes were the most represented (Fig. 4H).

For biological process analysis, most of the peptides were derived from biological regulation, cellular process, and metabolic process for RT and NBTXR3 + RT (Fig. 4I). As for previous analyses, we observed the emergence in NBTXR3 + RT of peptides whose biological process was not observed for RT alone (i.e., locomotion). Nonetheless, as the number of proteins in this process was fairly low (2 proteins), this did not substantially affect the overall proportions. However, there was a lower proportion of protein involved in the metabolic process for NBTXR3 + RT (20.6%) compared to RT alone (27%) (Fig. 4J–K). For RT vs. NBTXR3 + RT comparison, we can see that the obtained profile was very similar to NBTXR3 + RT (Fig. 4L).

Finally, for molecular function analysis, most of the peptides were derived from binding and catalytic activity for RT and NBTXR3 + RT (Fig. 4 M). In contrary to previous analyses, we observed the emergence in RT of peptides whose biological process was not observed for NBTXR3 + RT (i.e., molecular adaptor activity and translation regulator activity), but this represents only one protein for both categories. However, there was a greater proportion of molecular function regulator proteins for NBTXR3 + RT (12.3%) compared to RT alone (3%) (Fig. 4N–O). For RT vs. NBTXR3 + RT comparison, we can see that the obtained profile was globally comparable to NBTXR3 + RT (Fig. 4P).

**Fig. 5:**
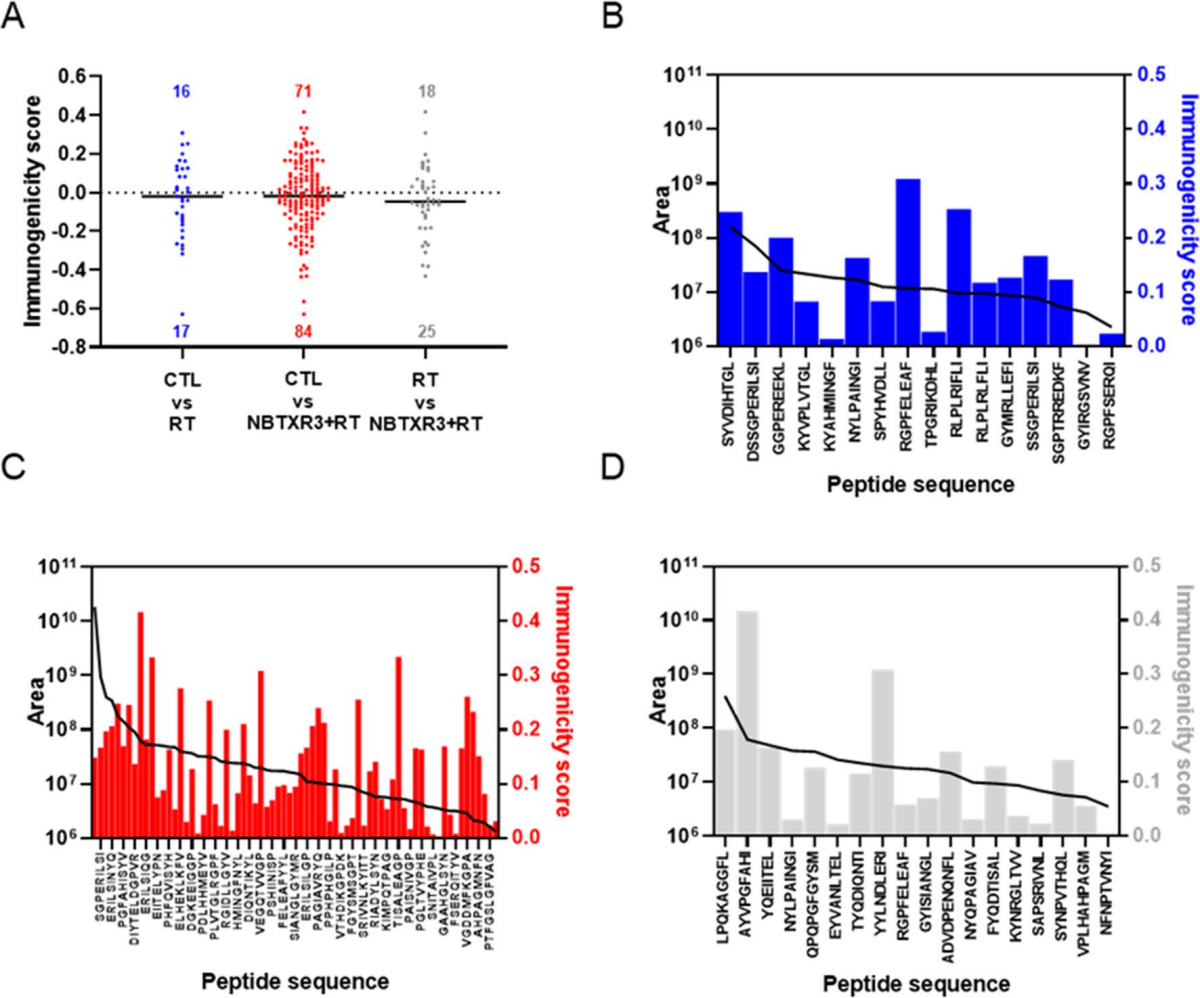
Immunogenicity score analysis. (**A**) Immunogenic score repartition of peptides with Log2 > 1 according to treatment and baseline comparator. Each dot represents one peptide. Barre, median. Immunogenic score (> 0) and corresponding area value for (**B**) CTL vs. RT, (**C**) CTL vs. NBTXR3 + RT and (**D**) RT vs. NBTXR3 + RT

We then evaluated the immunogenic potential of these peptides using the Class I Immunogenicity analysis tool [27] (Fig. 5). Thanks to the enhancement of peptide abundance triggered by NBTXR3 + RT, we can see that the number of peptides with a positive immunogenicity score was much more pronounced than for RT alone. Nonetheless, the median of immunogenicity scores was close to 0 for RT and NBTXR3 + RT, illustrating that the distribution between potentially immunogenic (> 0) and non-immunogenic (< 0) peptides was not modified by NBTXR3 (Fig. 5A). For RT vs. NBTXR3 + RT, a slight decrease in this median was observed. We then correlated the abundance of the peptides with their immunogenicity score (only for score > 0) (Fig. 5B–D). No direct relationship between the abundance of a peptide and the immunogenicity score was observed.

### NBTXR3 increases immune cell infiltrates

**Fig. 6:**
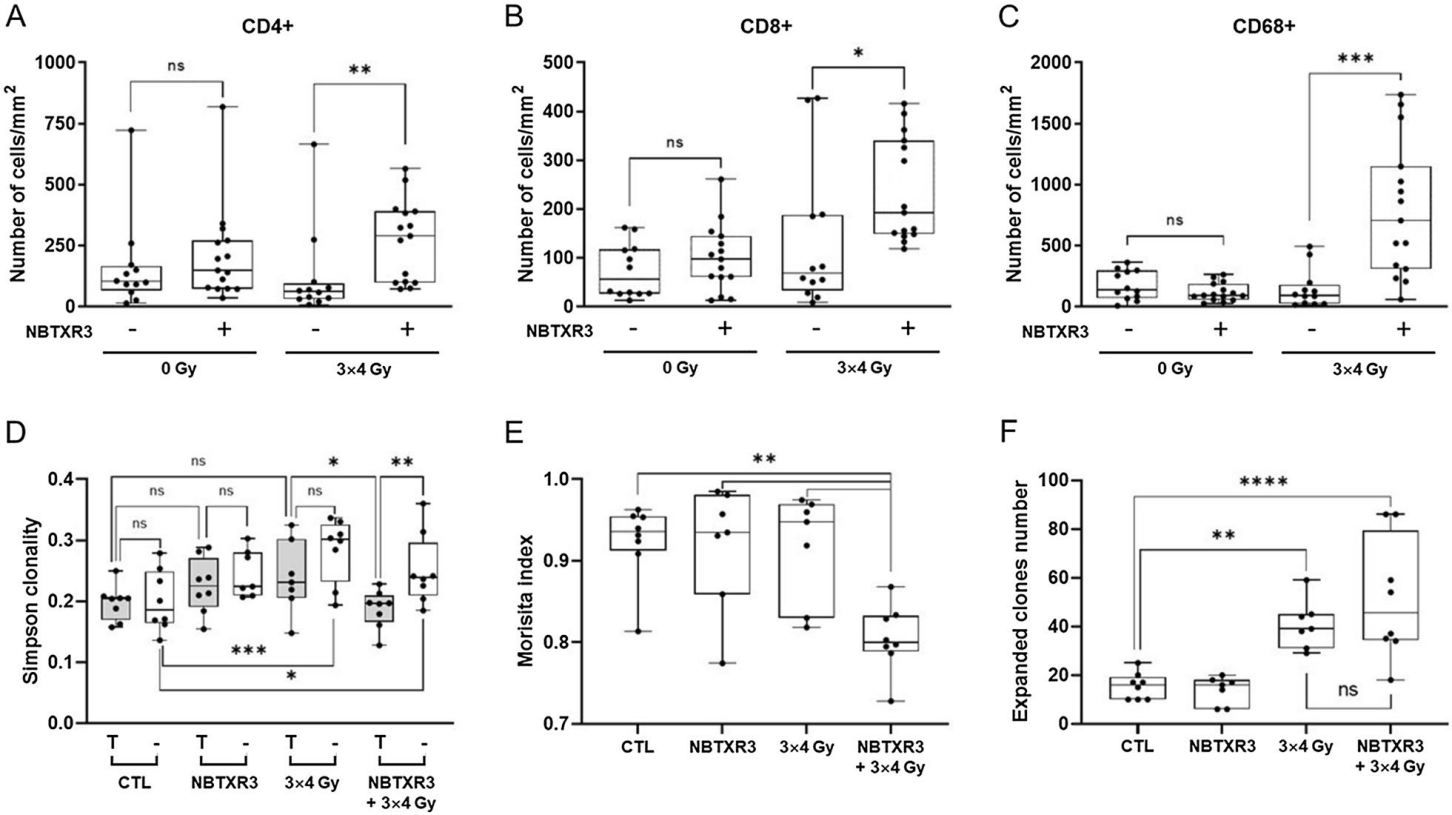
Modulation of TCR repertoire and immune cell infiltrates by NBTXR3 activated by RT. **A–C** Cell density of CD4+ (**A**), CD8+ (**B**) and CD68 + cells (**C**) infiltrates in treated tumors were analyzed by IHC, 5 days after the last fraction of RT. For each tumor, 3 slices from FFPE blocks (first third, middle and last third of the tumor) were put on the same slide and slices were stained using specific antibodies. Presented data were obtained from four to five mice per group (each dot represents one value). **D–F** Analysis of TCR repertoire. Box and whisker plot representations of Simpson clonality (**D**), Morisita index (**E**) and expanded clones (**F**) of TCR repertoire sequencing of treated (T) and untreated (–) tumors of CT26.WT bearing tumor mice, three days after the last fraction of RT. Presented data were obtained from seven to eight mice per group (each dot represents one value). Statistical test: One-Way ANOVA test for Simpson clonality and expanded clones, Mann-Whitney test for Morisita index. Bars represent the median. Statistical test: Mann-Whitney test. *p < 0.05; **p < 0.01; ***p < 0.001; ****p < 0.0001; ns, non-significant

We showed that NBTXR3 + RT could improve the immunogenicity of tumor cells through the modulation of their immunopeptidome and induction of ICD. We then evaluated the impact of this treatment on immune cell density in treated tumors. Single CT26.WT tumor-bearing immunocompetent mice were treated with or without NBTXR3, then irradiated (or not) with 4 Gy for 3 consecutive days. Five days after the last RT fraction, mice were sacrificed and CD4+, CD8+, and CD68 + cell densities in tumors were analyzed by digital pathology (Fig. 6A–C). For CD4 + T cells, we observed that the densities obtained for the control (no NBTXR3, no RT), NBTXR3 and RT groups were comparable, although a slight non-significant increase in this density was observed for NBTXR3, compared to the control (Fig. 6A). In stark contrast, a significant increase (*p <* 0.01) in CD4 + T cell density was measured in the group of mice treated with NBTXR3 + RT, compared to RT alone (median of CD4 + T cells/mm^2^: 290 and 62, for NBTXR3 + RT and RT, respectively). For CD8 + T cells, the densities obtained for control, NBTXR3, and RT alone groups were of the same order. Remarkably, we measured a significant increase (*p <* 0.05) for NBTXR3 + RT (median of CD8 + T cells/mm^2^: 193 and 69, for NBTXR3 + RT and RT, respectively) (Fig. 6B). A slight non-significant increase in CD8 + T cell density was observed for NBTXR3 compared to the control. There was a significant increase in CD68+ (macrophages) (*p <* 0.01) density only with NBTXR3 + RT (median of CD68 + cells/mm^2^: 707 and 94, for NBTXR3 + RT and RT, respectively). CD68 + cells represented the most abundant immune cell present in tumor tissues. Unlike with CD4 + and CD8 + densities, NBTXR3 had no perceptible effect on the density of CD68 + cells.

### NBTXR3 modulates the TCR repertoire

As NBTXR3 + RT modulated the immunopeptidome, we hypothesized that this could also impact TCR repertoire diversity. To verify this, we repeated the in vivo settings previously employed to demonstrate the abscopal effect produced by NBTXR3 + RT [21] and sequenced the TCR repertoire of treated and untreated tumors (Fig. 6D–F). We first analyzed the Simpson clonality, which reflects the TCR repertoire diversity (Fig. 6D). This unitless index ranges from 0 to 1, where values approaching 1 indicate a nearly monoclonal population. For the control and NBTXR3 groups, clonality was similar between the treated and untreated tumors. However, a slight not significant increase in the clonality value was observed with NBTXR3, compared to the control group. On the other hand, a fairly marked difference was observed between the treated and untreated tumors in the RT group, although this difference remained not significant (p = 0.07). Nonetheless, when untreated tumor results of RT and control groups were compared, the difference became strongly significant (*p <* 0.001). Interestingly, there was a significant difference between the treated and untreated tumors (*p <* 0.01) for the NBTXR3 + RT group. In addition, there was a significant difference between untreated tumors of this group vs. control (*p <* 0.05). Remarkably, a significant difference was also observed between the treated tumors of the RT and NBTXR3 + RT groups (*p <* 0.05).

We then assessed the level of similarity between TCR repertoires of treated and untreated tumors using the Morisita similarity index (Fig. 6E). This unitless index ranging from 0 to 1 considers the number of overlapping sequences between two TCR repertoires, where values approaching 1 indicate a near full similarity. Morisita index values for the control, NBTXR3, and RT groups were very similar and relatively close to 1 (median: 0.94, 0.93 and 0.95, respectively). Remarkably, a significant drop in the value of the Morisita index (*p <* 0.01) was observed for the NBTXR3 + RT group (median: 0.8), indicating the presence of heterogeneity between treated and untreated tumor TCR repertoires.

Finally, we measured the number of expanded clones in the treated tumors (Fig. 6F). Compared to control, the number of expanded clones for NBTXR3 was very similar. In stark contrast, a significant increase in expanded clones was observed for the RT and NBTXR3 + RT groups, compared to control (*p <* 0.01 and *p <* 0.0001, respectively). While the number of expanded clones appeared higher for the NBTXR3 + RT group, this trend was not significant compared to RT alone.

## Discussion

We previously reported that NBTXR3 + RT produced a significant CD8 + T cell-dependent abscopal effect in mice [21]. In addition, Hu et al. [22] recently published data showing that NBTXR3 + RT in combination with anti-PD-1 significantly improved both treated and distant untreated tumor control and survival, along with the induction of a sustained ATIR, in two mouse lung tumor models (sensitive or resistant to anti-PD-1 treatment). Taken together, these results illustrate the immunomodulatory capacity of NBTXR3 + RT. However, we currently have only limited information to understand the early-stage events that could lead to this potent ATIR. The ability of NBTXR3 + RT to destroy tumor cells more efficiently than RT alone has been demonstrated in a large panel of tumor cells, both in vitro and in vivo [15–17, 19]. Indeed, the increased amount of TAA/TSA released from dying cancer cells triggered by NBTXR3 + RT may improve the exposure of tumor antigens by dendritic cells, thereby stimulating the ATIR. However, other factors arising at various steps certainly come into play. Among the early factors, we postulated that NBTXR3 + RT could stimulate the ATIR through the modulation of tumor cell immunogenicity. To confirm this hypothesis, we first investigated the ability of NBTXR3 + RT to modulate calreticulin exposure on cancer cell surface membrane (also called ecto-calreticulin or Ecto-CALR), a biomarker of ICD [28, 29]. The translocation of calreticulin from the endoplasmic reticulum to the cell surface has been reported to be crucial for cancer cell immunogenicity and CD8 + mediated destruction [30, 31]. Here, we demonstrated in three cancer cell lines, including CT26.WT cells used for in vivo ‘abscopal’ experiments [21], that NBTXR3 + RT increased Ecto-CALR, compared to RT alone. We also measured an increase in ATP secretion and HMGB1 release in HCT116, 42-MG-BA and PANC-1 (see Supplemental Fig. 5). We also observed that NBTXR3 could increase Ecto-CALR without RT. The underlying mechanism remained unclear. Nonetheless, based on previous in vivo data with CT26.WT cells [21], as tumor growth was not affected by NBTXR3 alone, we can conclude that this signal was not sufficient to generate an efficient antitumor immune response. Taken together, these results support the hypothesis that NBTXR3 + RT may facilitate the ATIR through modification of the immunogenicity of cancer cells, as well as by induction of ICD.

The ‘immunopeptidome’ (the set of peptides presented by the MHC-I complex) plays a critical role in the immunogenicity of tumor cells [32]. Due to the peptide loaded in the MHC-I complex and specifically recognized by their TCR, CD8 + cytotoxic T lymphocytes can identify and destroy cancer cells. Based on this principle and previously reported results [21, 22], we evaluated the impact of NBTXR3 on the immunopeptidome profile and epitope abundance, using CT26.WT cells irradiated with or without NBTXR3. For cells treated only by NBTXR3, some variations were observed, but the overall immunopeptidome profile remained relatively similar to the control. Nonetheless, based on our previous in vivo studies showing that NBTXR3 did not affect tumor growth or immune cell infiltrates in treated tumors [21], we can conclude that these variations were not sufficient to generate an efficient antitumor immune response. On the other hand, RT globally promoted the abundance of peptides loaded in the MHC-I complex, when compared to control. This result is consistent with previous data published by Reits et al. [5] showing that RT can modulate the peptide repertoire of cancer cells. Remarkably, we measured a robust improvement of peptide abundance with NBTXR3 + RT. Thus, out of the 331 peptides systematically present whatever the condition considered, only 33 exhibited a Log2 > 1 for RT alone (i.e., approximately 10% of total peptides), versus 155 for NBTXR3 + RT (i.e., approximately 47% of total peptides). Interestingly, when compared to the RT immunopeptidome, 43 peptides were clearly over-represented and 10 less abundant for NBTXR3 + RT. Intriguingly, we observed the systematic decrease of MGPLKKDRI and SYALSRHDV peptide abundances, respectively related to RL3 and S35E1 proteins, with NBTXR3 (± RT). In addition, the peptides IYSEVATLI related to the protein PCM1 and SGPATHDI related to the protein were presented exclusively in cells treated by NBTXR3 + RT. No other ‘group-specific’ peptide has been observed. The biological significance of these observations and the underlying mechanisms remain unclear and require further investigation, but they seem to be related to NBTXR3.

We also investigated whether the effects of NBTXR3 + RT could go beyond the enhancement of epitope abundance, for example by impacting the “nature” of peptides presented by the MHC-I complex. To address this question, we first evaluated whether the non-homogeneous distribution of NBTXR3 in cells [15–17] could favor or prevent the presence of some epitopes in the immunopeptidome, depending on the intracellular location of the protein they come from. Variations in the proportions of the intracellular localization of the peptide-generating proteins were observed but they were relatively modest. This suggests that the “nature” of the immunopeptidome was not fundamentally influenced by the intracellular distribution of NBTXR3. The same conclusion could also be applied to protein class, biological process and molecular function analyzes. Regarding immunogenicity score, no fundamental changes induced by NBTXR3 have been observed. However, the overall improved immunopeptidome abundance combined with Ecto-CALR results show that NBTXR3 + RT modulated the immunogenicity of tumor cells. The enhanced abundance of peptides presented by MHC-I complex can promote weaker tumor epitope availability, thus enabling cytotoxic T lymphocyte responses against both dominant and subdominant TAA epitopes, as it has been previously reported for chemotherapy [33]. The immunogenicity score results (Fig. 5A) illustrate this possibility. In addition, these results are in agreement with those published by Gameiro et al. [30], showing that radiation-induced tumor immunogenic modulation promotes antigen processing and calreticulin exposure, facilitating tumor cell destruction by cytotoxic T lymphocytes.

This prompted us to compare the impact of the various treatments on the ATIR in the treated tumor. IHC analysis showed a significant increase of CD4 + T cells, CD8 + cytotoxic T cells and macrophage densities in the tumor treated with NBTXR3 + RT. In stark contrast, no effect of RT has been observed. These results are in agreement with our previous study and the production of a CD8 + T cell-dependent abscopal effect [21], confirming the potent immunomodulatory potential of NBTXR3 + RT. The impact on lymphocytes and previously reported abscopal effect [21] prompted us to investigate the effect of NBTXR3 + RT on TCR repertoire, both in treated and untreated tumors. We reported a significant widening of the TCR repertoire diversity in tumors treated with NBTXR3 + RT, compared to RT alone. This is consistent with published results using the mouse lung cancer 344SQ_R anti-PD-1 resistant cell line [22]. We also showed a significant difference in TCR repertoire diversity between the treated and untreated tumors of the NBTXR3 + RT group. Interestingly, we also observed a significant decrease in TCR overlapping between treated and untreated tumors only for NBTXR3 + RT, which could reflect the establishment of an abscopal response. Of note, there was also a significant increase in the number of expanded clones both for RT and NBTXR3 + RT, indicating that RT also had an impact on ATIR. Nonetheless, based on IHC results from our previous published data [21], the impact of RT was not strong enough to produce an efficient ATIR.

## Conclusions

Our results provide a better understanding of the biological responses triggered by RT-activated NBTXR3 within cancer cells. Fabian et al. [9] recently introduced the concept of ‘immunogenic cell stress’, regrouping both ICD and immunogenic modulations in the same continuum of biological pathways generated by chemotherapies, and leading to ATIR activation. This concept can also be applied to NBTXR3 + RT. Based on previous observations in non-clinical cancer models and the results presented here, it is clear that the effects of RT-activated NBTXR3 go beyond the ‘simple’ radio-enhancing ability previously reported [15–17]. Indeed, our results show that NBTXR3 activated by RT also acts as a potent immunomodulator at the cancer cell level, inducing an immunogenic cell stress strong enough to produce an effective ATIR. To our knowledge, this is also the first demonstration that high-Z radioenhancer nanoparticles can modulate cancer cell immunogenicity through the modification of the immunopeptidome. Taken together, these data emphasize the great potential of NBTXR3 activated by RT to become a key player in the radio-immuno-oncology field, opening new therapeutic opportunities to treat patients.

## Supplementary Information

**Additional file 1: Figure S1.** Histogram distribution of peptide length.** Figure S2.** Unique cellular component origins of proteins. For this analysis, only proteins originating from nucleus, cytosol, mitochondria, and Golgi apparatus were considered.** Figure S3.** Multiple cellular component origins of peptides.** Figure S4.** Protein class origins of proteins. For this analysis, only categories present in CTL vs RT were considered for CTL vs NBTXR3+RT.** Figure S5.** Extracellular ATP analysis for (A) HCT116, (B) 42-MG-BA and (C) PANC-1 cells. HMGB1 release measurement for (D) HCT116, (E) 42-MG-BA and (F) PANC-1 cells. Presented data were obtained from at least two independent experiments (n≥2). Data are represented as mean fold increase ± SEM compared to unirradiated control cells.** Table S1.** List of peptides and accession number of corresponding proteins.** Table S2.** NBTXR3 concentration (µM), irradiation dose (Gy), irradiation source and number of individual experiments for each cell line and each DAMP.

## Data Availability

Not applicable.

## Abbreviations

ATIR: Anti-tumor immune response
CTL: Control
DSB: Double-strand break
Ecto-CALR: Ecto-calreticulin
Gy: Gray
ICD: Immunogenic cell death
ROS: Reactive oxygen species
RT: Radiotherapy
TAA: Tumor-associated antigen
TSA: Tumor-specific antigen
