# Supplementary material for: Radiotherapy-activated NBTXR3 nanoparticles modulate cancer cell immunogenicity and TCR repertoire
Source: Cancer Cell Int. 2022 Jun 3;22:208. doi: 10.1186/s12935-022-02615-w (PMC9164428; doi:10.1186/s12935-022-02615-w)
Supplement: Supplementary file 1 — Additional file 1: Figure S1. Histogram distribution of peptide length. Figure S2. Unique cellular component origins of proteins. For this analysis, only proteins originating from nucleus, cytosol, mitochondria, and Golgi apparatus were considered. Figure S3. Multiple cellular component origins of peptides. Figure S4. Protein class origins of proteins. For this analysis, only categories present in CTL vs RT were considered for CTL vs NBTXR3+RT. Figure S5. Extracellular ATP analysis for (A) HCT116, (B) 42-MG-BA and (C) PANC-1 cells. HMGB1 release measurement for (D) HCT116, (E) 42-MG-BA and (F) PANC-1 cells. Presented data were obtained from at least two independent experiments (n≥2). Data are represented as mean fold increase ± SEM compared to unirradiated control cells. Table S1. List of peptides and accession number of corresponding proteins. Table S2. NBTXR3 concentration (µM), irradiation dose (Gy), irradiation source and number of individual experiments for each cell line and each DAMP. [file 12935_2022_2615_MOESM1_ESM.docx]

**Supplemental material**

**Cell line**

The human pancreatic cancer PANC-1 (CRL-1469) cell lines were purchased from the ATCC. Cells were cultivated according to provider’s recommendations.

**ATP Secretion and HMGB1 release analysis**

For extracellular ATP levels measurement, the culture medium was collected and analyzed for ATP content using the luciferin-based ENLITEN® ATP assay (Promega), 9h after irradiation. ATP-driven chemoluminescence was recorded on a Glomax 96 microplate luminometer (Promega). Extracellular release of HMGB1 in the culture medium was assessed 72h to 96h hours after irradiation by an ELISA assay (IBL-international). Optical density was recorded on a Powerwave 340 spectrometer (BioTek). NBTXR3 concentration, irradiation dose, irradiation source and the number of individual experiments for each cell line are reported in Supplemental Table 2.

**Supplemental figures**


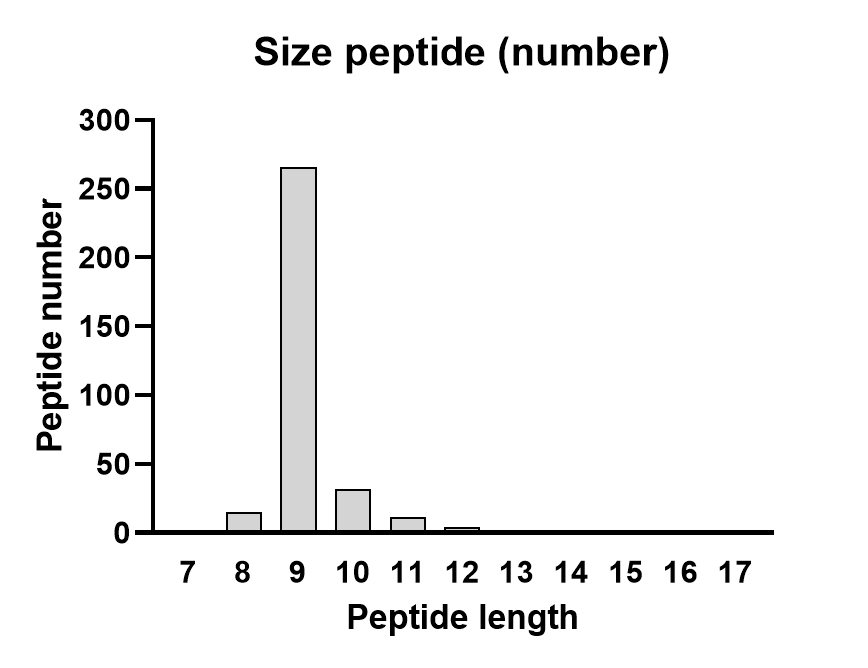


**Supplemental Figure 1**: Histogram distribution of peptide length.


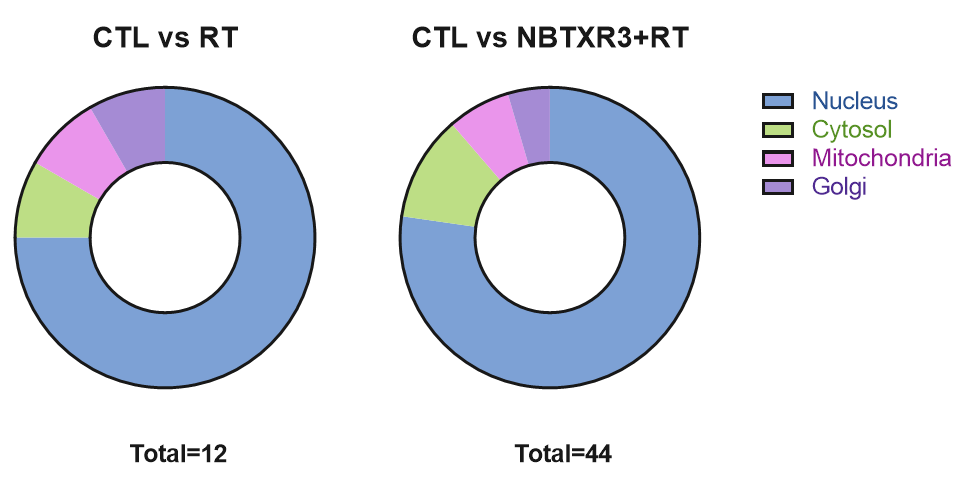


**Supplemental Figure 2**: Unique cellular component origins of proteins. For this analysis, only proteins originating from nucleus, cytosol, mitochondria, and Golgi apparatus were considered.


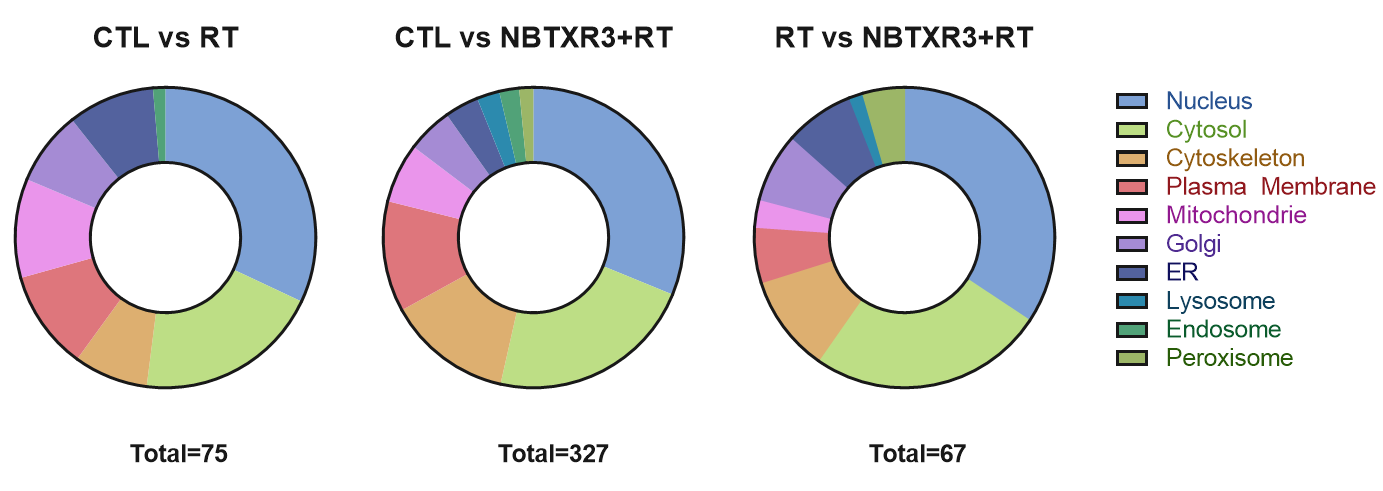


**Supplemental Figure 3**: Multiple cellular component origins of peptides.


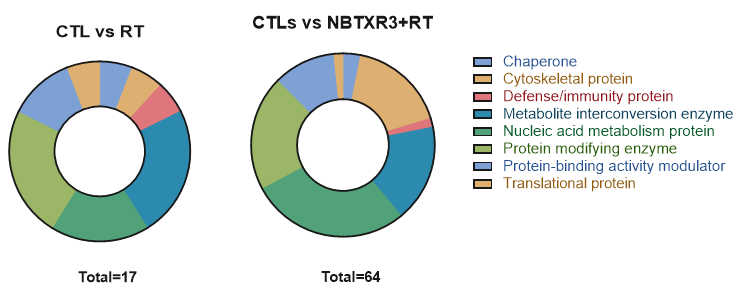


**Supplemental Figure 4:** Protein class origins of proteins. For this analysis, only categories present in CTL vs RT were considered for CTL vs NBTXR3+RT.


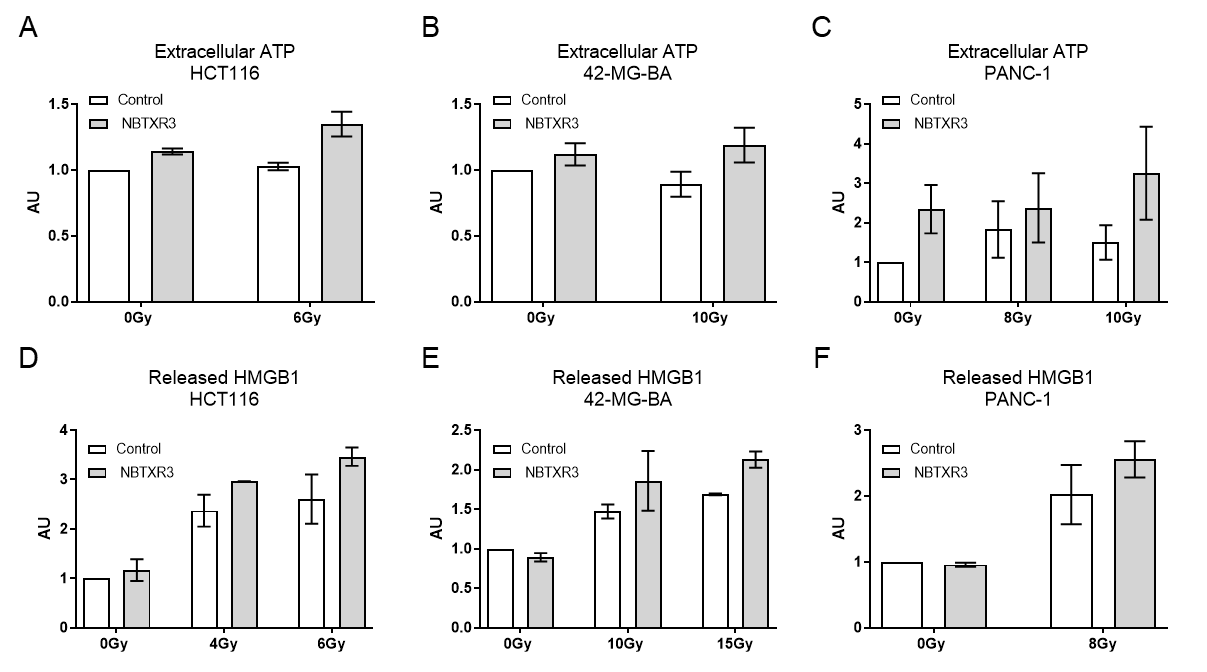


**Supplemental Figure 5**: Extracellular ATP analysis for (**A**) HCT116, (**B**) 42-MG-BA and (**C**) PANC-1 cells. HMGB1 release measurement for (**D**) HCT116, (**E**) 42-MG-BA and (**F**) PANC-1 cells. Presented data were obtained from at least two independent experiments (n≥2). Data are represented as mean fold increase ± SEM compared to unirradiated control cells.

**Supplemental table**

**Supplemental Table 1**: List of peptides and accession number of corresponding proteins.

**Supplemental Table 2**: NBTXR3 concentration (µM), irradiation dose (Gy), irradiation source and number of individual experiments for each cell line and each DAMP.
